# Reverse remodeling after coronary revascularization in patients with ischemic heart disease: focused on change of infarct scar

**DOI:** 10.1186/1532-429X-15-S1-P183

**Published:** 2013-01-30

**Authors:** Eun-Ah Park, Whal Lee, Yeo Koon Kim, Jin Wook Chung

**Affiliations:** 1 Radiology, Seoul National University Hospital, Seoul, Republic of Korea

## Background

We sought to evaluate the serial change of enhanced necrotic tissue and nonenhanced noninfarct tissue after surgical revascularization in patients with ischemic left ventricular (LV) dysfunction using magnetic resonance (MR) imaging as a quantification tool.

**Figure 1:**
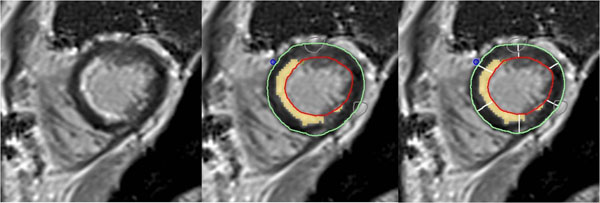


**Figure 2:**
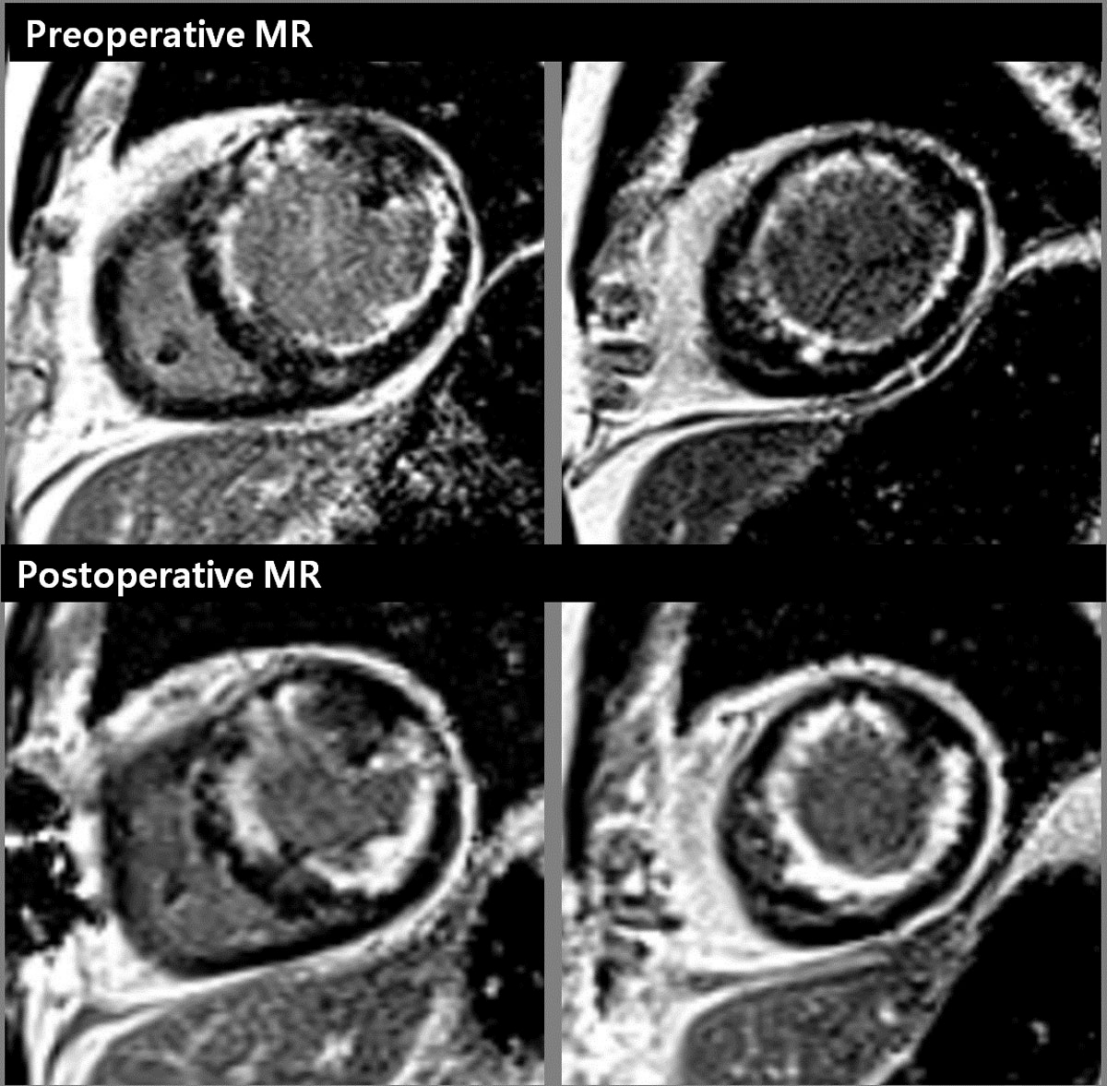


## Methods

Twenty-four patients with severe LV dysfunction {ejection fraction (EF) <35% on echocardiography} underwent before and more than 6 months after surgical revascularization. The following variables were measured: LV volumes, EF, mass, absolute and relative infarction size, transmural extent of infarction, and wall thickness. All patients were classified into two groups: group I, presence of revere remodeling after revascularization; group II, absence of reverse remodeling group. A reduction of 10% or more in end-diastole and end-systole volumes were considered presence of reverse remodeling. Variables were compared between preoperative and postoperative MR scans in each group.

## Results

Of 24, 20 patients showed reverse remodeling. In group I, EF (28.8±6.6% vs. 40.6±7.8, p<0.0001) was significantly improved. Group I showed no change of absolute infarction size (17.3±10.9 g vs. 17.5±10.4 g, p=0.8) but significant increase in relative infarction size (21.0±13.7% vs. 26.5±19.4%, p=0.01) because of reduction of myocardial mass after revascularization. Significant increase in regional transmural extent (35.2±22.8% vs. 39.6±24.2%, p<0.0001) and thickness of enhanced tissue (4.2±1.5 mm vs. 5.9±1.8 mm, p<0.0001) were found in group I. However, thicknesses of nonenhanced rim and remote myocardium were not changed. There were no significant changes in all variables in group II.

## Conclusions

MR imaging demonstrated the significant increase in depth of necrotic tissue during the reverse remodeling. It can be explained by reverse slippage of the necrotic tissue. Transmural extent of necrotic tissue in patients with severe LV remodeling may be underestimated, resulting in false positive interpretation of viability.

## Funding

none

